# Non-coding RNA-mediated tug-of-war between emerging viruses and host antiviral defense: lessons from animal viruses

**DOI:** 10.1080/22221751.2026.2746261

**Published:** 2026-10-07

**Authors:** Na Chen, Baoge Zhang, Ziding Yu, Guangzhi Tong, Jiayu Jin, Qi Jiang, Xiaoyong Chen

**Affiliations:** a College of Life Sciences, Zhejiang Normal University, Jinhua, People’s Republic of China; b Department of Pathogen Biology, School of Basic Medical Sciences, Fujian Medical University, Fuzhou, People’s Republic of China; c Key Laboratory of Gastrointestinal Cancer (Fujian Medical University), Ministry of Education, Fuzhou, People’s Republic of China; d MOE Joint International Research Laboratory of Animal Health and Food Safety, Engineering Laboratory of Animal Immunity of Jiangsu Province, College of Veterinary Medicine, Nanjing Agricultural University, Nanjing, People’s Republic of China; e Fujian Academy of Chinese Medical Sciences, Fuzhou, People’s Republic of China; f College of Food and Bioengineering, Wuhu Vocational Technical University, Wuhu, People’s Republic of China; g Shanghai Veterinary Research Institute, Chinese Academy of Agricultural Sciences, Shanghai, People’s Republic of China; h College of Veterinary Medicine, China Agricultural University, Beijing, People’s Republic of China; i Institute of Pathogen Biology, Chinese Academy of Medical Sciences and Peking Union Medical College, Beijing, People’s Republic of China

**Keywords:** Non-coding RNA, animal virus-host interactions, antiviral innate immunity, viral hijacking, viral antagonism, antiviral drug targets

## Abstract

Emerging animal viruses pose persistent threats to livestock breeding and public health, underscoring the need for a deeper understanding of virus-host interactions. Nevertheless, the regulatory roles and conserved mechanisms of host non-coding RNAs (ncRNAs) during animal virus infections remain fragmented, and ncRNA-mediated mechanisms for emerging animal viruses are particularly poorly characterized, with no systematic collation or theoretical summarization available. Studies indicate that ncRNAs, including miRNAs, lncRNAs, and circRNAs, have emerged as important regulators of the interplay between animal viruses and their hosts. This review synthesizes evidence from eleven animal viruses covering swine, poultry, ruminant, feline, canine, and mink hosts, with emphasis on virus-induced changes in ncRNA expression and their antiviral or proviral functions. Across diverse virus-host systems, three recurring regulatory paradigms are identified: host antiviral defense, viral hijacking, and viral antagonism. Host cells can dynamically reshape their ncRNA expression to regulate host restriction factors, proviral host factors, and other ncRNAs, thereby influencing innate immune signalling, viral replication cycles, autophagy, apoptosis, and metabolic reprogramming. Conversely, animal viruses, including PEDV, PRRSV, PRV, SVA, AIV, ALV-J, NDV, BVDV, FHV-1, CIV, and MEV, have evolved sophisticated strategies to hijack host ncRNA networks or antagonize ncRNA-mediated antiviral immunity, and remodel the intracellular microenvironment to facilitate viral replication. These paradigms provide a unified theoretical system for understanding and predicting ncRNA-mediated regulatory mechanisms in emerging animal viruses. They also offer references for dissecting the pathogenic patterns of ASFV, SADS-CoV, and future unknown emerging viruses, as well as, for mining, screening, and identifying potential antiviral drug targets.

## Glossary

AIV: Avian influenza virus
Alix: ALG-2-interacting protein X
ALV-J: Avian leukosis virus subgroup J
ASFV: African swine fever virus
ATG9A: Autophagy-related protein 9A
ATG14: Autophagy-related protein 14
BVDV: Bovine viral diarrhea virus
CD163: Cluster of differentiation 163
CEFs: Chicken embryonic fibroblasts
ceRNA: Competing endogenous RNA
cGAS: Cyclic GMP-AMP synthase
CircRNAs: Circular RNAs
CIV: Canine influenza virus
CLDN4: Claudin-4
c-Jun: Cellular Jun
CNTN1: Contactin 1
CREBBP: CREB-binding protein
CYLD: Cylindromatosis protein
DDX3X: DEAD-box helicase 3 X-linked
DDX6: DEAD-box helicase 6
DEFs: Duck embryo fibroblasts
DNMT1: DNA methyltransferase 1
DR1: Down-regulator of transcription 1
DUSP1: Dual specificity phosphatase 1
ELAVL4: ELAV-like protein 4
FAM131C: Family with sequence similarity 131 member C
FAM134B: Family with sequence similarity 134 member B
FGF2: Fibroblast growth factor 2
FHV-1: Feline herpesvirus 1
GAB3: GRB2-associated binding protein 3
HDACs: Histonedeacetylases
HNRNPA3: Heterogeneous nuclear ribonucleoprotein A3
HO-1: Heme oxygenase 1
HSP60: Heat shock protein 60
IFITs: Interferon-induced proteins with tetratricopeptide repeats
IFITM1: Interferon-induced transmembrane protein 1
IFN-α/β: Interferon α/β
IFNAR: Interferon alpha/beta receptor
IRAKs: Interleukin-1 receptor-associated kinases
IRFs: Interferon regulatory factors
ISGs: IFN-stimulated genes
ISRE: IFN-stimulated response elements
JAK1: Janus kinase 1
JNK: c-Jun N-terminal kinase
KEAP1: Kelch-like ECH-associated protein 1
LIF: Leukemia inhibitory factor
LncRNAs: Long non-coding RNAs
LNP: Lipid nanoparticle
MAPK8: Mitogen-activated protein kinase 8
MAVS: Mitochondrial antiviral signaling
MDA5: Melanoma differentiation-associated gene 5
MEV: Mink enteritis virus
miRNAs: MicroRNAs
Mx1: Myxovirus resistance 1
NcRNAs: Noncoding RNAs
NDV: Newcastle disease virus
NEDD4/9: Neuronal precursor cell expressed developmentally downregulated 4/9
NF-κB: Nuclear factor κappa-light-chain-enhancer of activated B cells
NFIA: Nuclear factor I A
OAS2: 2'-5'-oligoadenylate synthase 2
OASL: 2'-5'-oligoadenylate synthetase-like protein
PAMs: Porcine alveolar macrophages
PEDV: Porcine epidemic diarrhea virus
PI3K: Phosphatidylinositol 3-kinase
PKC: Protein kinase C
PKR: Protein kinase R
PP2A: Protein phosphatase 2A
PRRSV: Porcine reproductive and respiratory syndrome virus
PRV: Pseudorabies virus
RACK1: Receptor for activated C kinase 1
RIG-I: Retinoic acid-inducible gene-I
RISC: RNA-induced silencing complex
RNF11: Ring finger protein 11
RSAD2: Radical S-adenosyl methionine domain-containing protein 2
RXRB: Retinoid X receptor β
SADS-CoV: Swine acute diarrhea syndrome coronavirus
SOCSs: Suppressors of cytokine signalling
SREBF1: Sterol regulatory element-binding transcription factor 1
SRP14: Signal recognition particle 14 kDa protein
STATs: Signal transducers and activators of transcriptions
SVA: Senecavirus A
TBK1: TANK-binding kinase 1
TfR: Transferrin receptor
TLR3/7: Toll-like receptor 3/7
TRAF3: TNF receptor-associated factor 3
TRIMs: Tripartite motif proteins
USP25: Ubiquitin specific peptidase 25
ZMYND11: Zinc finger MYND domain-containing protein 11
ZNF34/135: Zinc finger protein 34/135
ZO1: Zonula occludens protein 1

## Introduction

Animal virus infections pose a major threat to global livestock breeding and public health. Porcine viruses, including porcine epidemic diarrhea virus (PEDV), porcine reproductive and respiratory syndrome virus (PRRSV), pseudorabies virus (PRV), Senecavirus A (SVA), African swine fever virus (ASFV), and swine acute diarrhea syndrome coronavirus (SADS-CoV), avian viruses represented by highly pathogenic H5/H7 subtype avian influenza virus (AIV), avian leukosis virus subgroup J (ALV-J), and Newcastle disease virus (NDV), as well as cross-species viruses including bovine viral diarrhea virus (BVDV), feline herpesvirus 1 (FHV-1), canine influenza virus (CIV), and mink enteritis virus (MEV), can result in substantial economic losses and biosafety concerns through efficient replication, immune evasion, and cross-host transmission [1, 2]. The outcome of viral infection depends on the dynamic interplay between viruses and hosts. As the first line of defense against viral invasion, host innate immunity activates multilayered signalling pathways and effector mechanisms. In response, viruses have evolved two core strategies during long-term co-evolution: hijacking host regulatory networks and antagonizing host antiviral defense. They reshape the intracellular microenvironment by manipulating key host molecules to achieve immune evasion and establish viral replication [1, 3–5].

Host non-coding RNAs (ncRNAs), including microRNAs (miRNAs), long non-coding RNAs (lncRNAs), and circular RNAs (circRNAs), are a class of functionally critical regulatory molecules without protein-coding capacity, and have been validated as important hubs governing host antiviral defense and viral replication [1, 4]. During animal virus infection, host ncRNAs can undergo substantial changes in abundance and activity and regulate antiviral responses by targeting key nodes such as innate immune signalling pathways, viral replication cycles, autophagy, apoptosis, and metabolic reprogramming. On the host side, induction of antiviral ncRNAs can directly inhibit viral replication or amplify immune antiviral signalling [6–8], while repression of proviral ncRNAs can relieve inhibitory effects on host defense pathways [9, 10]. Conversely, animal viruses can manipulate host ncRNAs: upregulating proviral ncRNAs to achieve immune hijacking, or downregulating antiviral ncRNAs to execute immune antagonism, thereby weakening host defenses and creating a replication-permissive cellular environment [11, 12]. Accumulating evidence highlights that host ncRNAs serve as promising molecular targets for antiviral intervention, providing new avenues for the development of novel antiviral strategies [3, 5]. A hierarchical schematic (Figure 1) is presented to clarify the organizational logic of this review. It delineates two critical dimensions governing ncRNA-mediated host-virus crosstalk and identifies three primary regulatory paradigms: host antiviral defence, viral hijacking, and viral antagonism.

**Figure 1.**
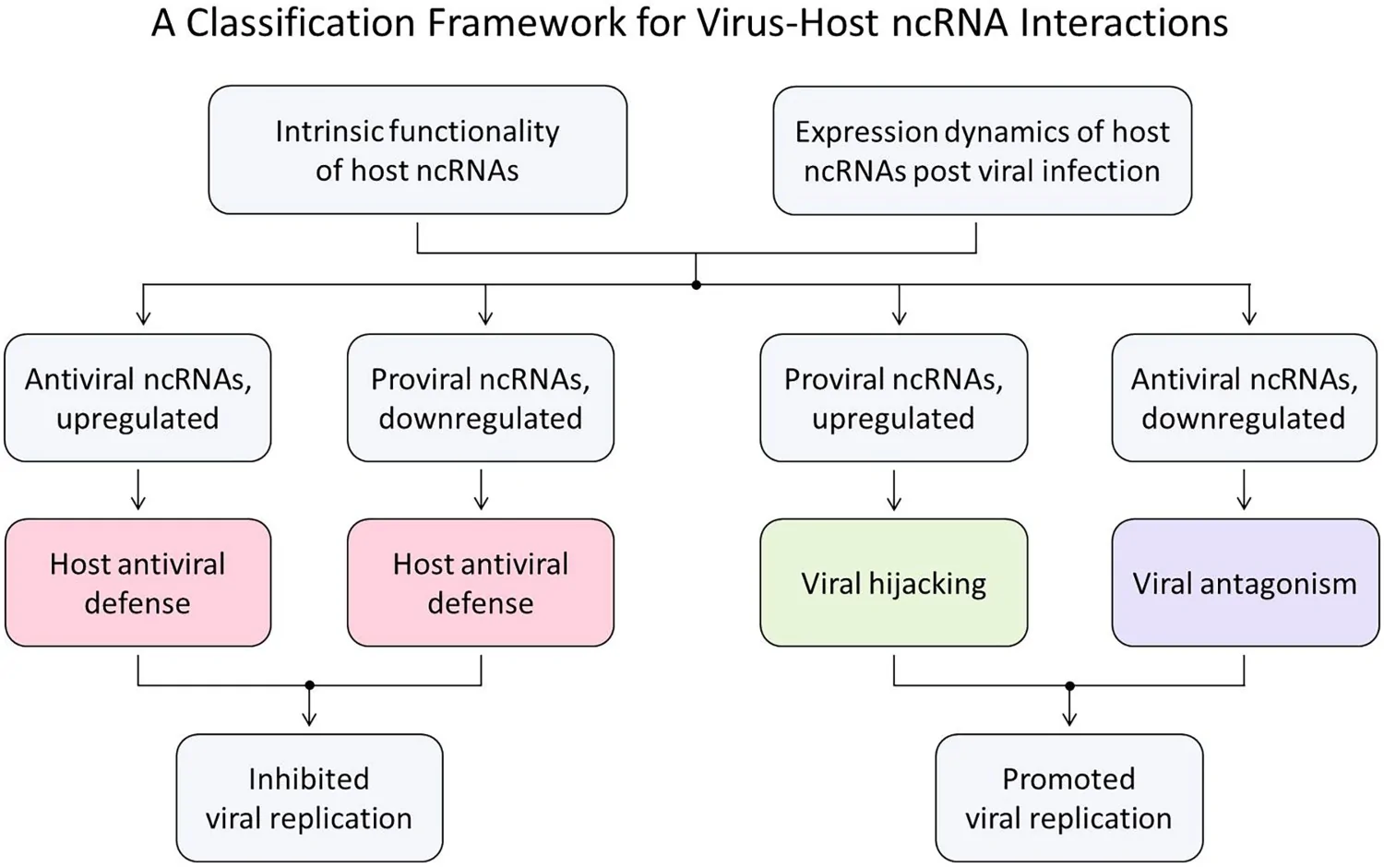
Hierarchical classification framework illustrating virus-host ncRNA crosstalk. Two foundational dimensions, the intrinsic biological function of host ncRNAs and their expression dynamics after viral infection, jointly determine three core regulatory paradigms, namely host antiviral defense, viral hijacking, and viral antagonism. The combined outcome of these interactions either inhibits or promotes viral replication.

Although research on ncRNA-mediated interplay between animal viruses and their hosts has expaned rapidly, the underlying mechanisms remain fragmented across individual virus-host systems, and an integrated framework spanning multiple species and viral taxa is still lacking [7, 13–15]. Most studies focus on the function of a single ncRNA in a specific virus, and fail to integrate infection-induced expression patterns (upregulation/downregulation) with regulatory effects (antiviral/proviral), making it difficult to reveal conserved cross-viral regulatory principles. Moreover, ncRNA-mediated mechanisms in critical emerging viruses such as ASFV and SADS-CoV remain poorly characterized, hindering predictive research for emerging and unknown viruses based on known regulatory paradigms. Therefore, systematically summarizing the conserved regulatory principles of ncRNAs during animal virus infection and establishing a unified interaction framework have become urgent scientific issues for dissecting pathogenic mechanisms and identifying broad-spectrum intervention targets.

Within this review, emerging viruses are defined as viruses that are newly-emergent, generated via genomic reassortment, or cause large-scale outbreaks after key genetic variation. This review systematically compiles experimental evidence of host ncRNA-virus interactions from eleven animal viruses, including PEDV, PRRSV, PRV, SVA, AIV, ALV-J, NDV, BVDV, FHV-1, CIV, and MEV. Within this panel of eleven viruses analyzed in the present study, SVA and highly pathogenic H5/H7-subtype AIV serve as representatives of emerging viruses, while the remaining viruses are well-established animal pathogens with sufficient accumulated research data [16]. On the basis of published experimental results from this panel of viruses, we distill cross-virus conserved regulatory paradigms. The resulting paradigms can be applied to multiple research scenarios. They can realize mechanistic supplementation and expansion of research thinking for SVA and AIV, which possess a partial research basis for ncRNA-related mechanisms. They can provide predictive research reference for ASFV and SADS-CoV, for which virus-host ncRNA interaction mechanisms remain entirely blank. They can also deliver paradigm-level research strategies and target-screening ideas for yet-unknown emerging animal viruses that may arise in the future [17].

This review synthesizes evidence on virus-induced ncRNA expression and functional mechanisms across more than 10 animal viruses spanning swine, poultry, ruminant, feline, canine, and mink hosts. We organize the available evidence into three recurring regulatory paradigms: host antiviral defense, viral hijacking of host ncRNA networks, and viral antagonism of ncRNA-mediated antiviral responses, which encompass host upregulation of antiviral ncRNAs, host downregulation of proviral ncRNAs, viral hijacking of proviral ncRNAs, and viral antagonism of antiviral ncRNAs. We further systematically collate and extract several candidate targets with broad-spectrum regulatory potential across diverse animal viruses (such as miR-155, miR-181, miR-203, miR-26a, and miR-30). Compared with previous reviews that largely focus on a single species, a limited set of viruses, and merely enumerate ncRNA functions in a fragmented manner, this review makes incremental contributions in virus-coverage scope, expression-function integration of ncRNAs, cross-species generalization, and construction of conserved paradigms (see Table 1 for details). While the ncRNA-mediated mechanisms of ASFV, SADS-CoV, and currently unknown emerging animal viruses remain largely elusive, critical lessons and predictive insights can be drawn from the conserved paradigms summarized in this review, providing a unified theoretical framework for rapidly dissecting pathogenic mechanisms and efficiently screening intervention targets. This review not only systematically improves the molecular networks of ncRNAs-mediated interplay between animal viruses and hosts but also offers an important theoretical basis for the mechanistic dissection and development of broad-spectrum antiviral strategies against emerging animal viruses by synthesizing key facets of this ongoing tug-of-war.

**Table 1.** Comparison of incremental contributions between this review and previously-published related reviews.

No.	Comparison dimension	Previously-published reviews in the field	Innovative features of this review
1	Integrative logic for linking ncRNA expression and function	Prior reviews only dissect the regulatory functions of ncRNAs in viral replication, without systematically associating post-infection ncRNA expression changes with pro-/antiviral effects, and they lack a unified logical framework.	Establishes for the first time a linked analytical framework connecting infection-induced expression alterations and biological regulatory functions governing viral replication. By using panoramic framework diagrams (Figure 1) and mechanistic schematics (Figures 2–5), this review interconnects ncRNA expression patterns, regulatory effects, and the three major conserved paradigms, and visually illustrates the three-layer core regulatory logic underlying virus-host tug-of-war.
2	Organizational logic of manuscript sections	Most prior reviews organize sections by ncRNA category or by the ncRNA-dependent regulatory mechanisms governing viral replication.	This review organizes manuscript sections centered on the three conserved regulatory paradigms for the first time, constructing a narrative system for virus-host tug-of-war and breaking conventional writing frameworks based on ncRNA categories or individual ncRNA functions.
3	Informational design of mechanistic schematics	Mechanistic diagrams in the vast majority of reviews merely display ncRNAs and their regulation of downstream targets, without annotating post-infection expression shifts of ncRNAs themselves; therefore, readers cannot distinguish between host-dominated and virus-dominated regulatory programs.	All mechanistic schematics concurrently annotate post-infection expression changes of ncRNAs, enabling intuitive discrimination among three modes: host antiviral defense, viral hijacking and viral antagonism, and improving the readability and logical integrity of the paradigms.
4	Informational dimensions of summary tables	Summary tables in prior reviews fail to clearly display the regulatory relationships corresponding to the three conserved paradigms.	An additional column is introduced in Tables 2 and 3 to assign entries to the three conserved paradigms. Post-infection ncRNA expression changes and their regulatory effects on viral replication are annotated synchronously, achieving logical closure among the main text, mechanistic schematics and summary tables.
5	Breadth of coverage of viruses and ncRNA molecules	Prior reviews analyze one or multiple classes of ncRNAs with a focus on a single virus, or characterize one ncRNA class across only a small set of viruses. They lack systematic integration across multiple viruses and all three major classes of host ncRNAs.	Systematically compiles research evidence from 10 representative animal viruses and summarizes regulatory functions of the three major classes of host ncRNAs, achieving systematic integration of regulatory data across multiple viruses and multiple ncRNA classes.
6	Scope of cross-species generalization across host organisms	Prior reviews are mostly restricted to a single animal species, and fail to systematically extract cross-species conserved rules.	This review synthesizes experimental evidence of viral infection across multiple hosts including swine, poultry, ruminants, felines, canines, and mink, distills conserved ncRNA-dependent regulatory rules across species, and provides generalized theoretical references for emerging animal viruses.
7	Predictive value of the theoretical framework	Prior reviews only make scattered remarks regarding emerging viruses, and do not systematically elaborate how synthesized theoretical frameworks can guide research on emerging viruses.	Based on the three distilled conserved interaction paradigms, this review lays a theoretical foundation for understanding and predicting ncRNA-mediated regulatory mechanisms in emerging animal viral infections.

## NcRNA-mediated host antiviral defense

### Induction of host antiviral ncRNAs

In response to animal virus infection, host cells rapidly induce a diverse repertoire of antiviral ncRNAs, thereby initiating multilayered innate immune responses and directly restricting viral proliferation. Infections with porcine viruses trigger robust upregulation of miRNAs, lncRNAs, and circRNAs targeting proviral host factors or directly inhibit viral genomes. Upon PEDV infection, exosomal miR-1343 is markedly induced in porcine intestinal epithelial cells. As an antiviral miRNA, miR-1343 directly targets the 3′UTR of FAM131C to downregulate its expression. FAM131C acts as a proviral factor by suppressing host innate immune signalling and promoting PEDV replication. Consequently, exosomally delivered miR-1343 inhibits viral proliferation by repressing FAM131C and restoring antiviral immune responses, highlighting an important host defense mechanism against PEDV infection [6]. Similarly, PEDV infection significantly upregulates host miR-221-5p expression, which exerts dual antiviral effects. On one hand, it directly targets the highly conserved 3′UTR of the PEDV genomic RNA, thereby inhibiting viral replication. On the other hand, it activates the NF-κB signalling pathway. This miRNA directly targets and downregulates NFKBIA (IκBα) and SOCS1, two negative regulators of innate immunity, thereby enhancing the production of IFN-α/β and downstream antiviral ISGs, including ISG15 and Mx1 [13]. Beyond miRNAs, PEDV infection also significantly upregulates host lncRNA-446 expression, which directly binds to Alix and inhibits its ubiquitination and degradation mediated by the E3 ubiquitin ligase TRIM25, thereby stabilizing Alix protein levels. Alix subsequently interacts with tight junction proteins ZO1 and occludin to maintain intestinal barrier integrity. Thus, lncRNA-446-mediated stabilization of Alix contributed to the suppression of PEDV replication [18]. For PRRSV, infection markedly downregulates host transcription factor IRF8, a negative regulator of miR-10a, relieving transcriptional repression and thereby upregulating miR-10a expression. miR-10a directly targets the 3′UTR of SRP14 and inhibits its expression, and SRP14 acts as a proviral factor that interacts with viral Nsp2 to promote PRRSV genomic RNA synthesis and replication, establishing the IRF8-miR-10a-SRP14 antiviral regulatory axis [19]. Highly pathogenic PRRSV infection also upregulates host miR-150 expression through activation of the PKC/JNK/c-Jun signalling pathway. miR-150 exerts dual antiviral effects by directly targeting the conserved 3′UTR of the viral genome to inhibit replication and by enhancing type I interferon signalling. This miRNA directly represses SOCS1, a key negative regulator of the JAK-STAT pathway. Notably, it exhibits broad-spectrum activity against other porcine RNA viruses such as PEDV [20]. In addition, PRRSV infection upregulates host miR-26a expression, which does not directly target the PRRSV genome but promotes the expression of IFN-α/IFN-β and downstream antiviral effectors, including Mx1 and ISG15, strongly inhibiting the replication of both genotype 1 and genotype 2 PRRSV strains in a dose-dependent manner [7]. Furthermore, PRRSV infection upregulates host miR-361-3p expression, which acts as a direct antiviral effector by specifically targeting the highly conserved ORF1a (Nsp11) and ORF1b (Nsp12) regions in the PRRSV genome, markedly suppressing viral gene transcription and reducing PRRSV titres [21]. Infection with either highly pathogenic or low-pathogenic PRRSV also upregulates host miR-c89 expression, which markedly suppresses the replication of multiple PRRSV strains by directly targeting the 3′UTR of porcine RXRB mRNA in a sequence-specific manner [22]. Moreover, PRRSV infection induces host lncRNA-000397 expression in PAMs, which negatively regulates PRRSV replication by promoting the expression of antiviral ISGs, including Mx1, ISG15, and RSAD2 [14]. For PRV, infection markedly elicits the expression of both host miR-155-5p and lncRNA-155 in vitro and in vivo. These two ncRNAs exert independent antiviral effects via distinct signalling cascades. miR-155-5p reinforces antiviral innate immunity by amplifying STAT1 phosphorylation and boosting the expression of downstream ISG15 and OAS2. In parallel, lncRNA-155 facilitates the production of IFN-β by enhancing the activation of IRF3, independently of miR-155-5p [23]. PRV infection also significantly upregulates host circRNA-29164 expression in PK-15 cells, which functions as a ceRNA that directly sponges miR-24-3p, thereby abrogating miR-24-3p-mediated translational repression of KEAP1. The elevated KEAP1 triggers mitochondrial cytochrome c release and caspase-3-dependent intrinsic apoptosis, restricting PRV replication, with this axis conserved against influenza A virus and porcine deltacoronavirus [24]. Together, these mechanisms establish ncRNAs as critical components of the host defense system against porcine viral pathogens.

In avian species, infection with AIV and NDV elicits distinct yet evolutionarily conserved antiviral ncRNA signatures. During H5N6 AIV infection, host miR-200c is markedly induced and directly targets the 3′UTRs of CNTN1 and USP25 to suppress their expression. CNTN1 interacts with MAVS and recruits USP25 to catalyze K63-linked deubiquitination of MAVS, leading to proteasomal degradation of MAVS and inhibition of RIG-I-mediated type I interferon signalling. miR-200c downregulates CNTN1 and USP25 to prevent K63-linked deubiquitination and proteasomal degradation of MAVS, thereby elevating the expression of ISG15, OASL, and IFIT1 and ultimately restricting H5N6 replication [15]. Similarly, H5N1 AIV infection upregulates host miR-203 expression via two independent mechanisms. Type I interferon signalling directly activates miR-203 transcription, and downregulation of DNMT1 drives DNA demethylation of the miR-203 promoter. miR-203 subsequently exerts antiviral activity by directly targeting the 3′UTR of DR1 and repressing its expression. DR1 functions as a proviral factor by suppressing host interferon production and promotes viral RNA-dependent RNA polymerase activity to facilitate H5N1 replication [25]. AIV, including highly pathogenic H5N1, H7N9, and H1N1 strains, significantly induces host lncRNA-45. Upon infection, it translocates from the nucleus to the cytoplasm and suppresses the replication of multiple AIV subtypes by inhibiting viral polymerase activity, limiting the nuclear accumulation of viral NP and PA proteins, and alleviating virus-induced apoptosis [26]. Likewise, AIV, including H5N1, H7N9, and multiple other subtypes, significantly upregulates host lncRNA-61, which translocates to the cytoplasm upon infection and exerts broad-spectrum antiviral activity against nearly all tested avian and human influenza A viruses by interfering with viral entry, RNA synthesis, and virion release, with LNP-encapsulated lncRNA-61 effectively suppressing viral replication in mice [27]. In addition, H9N2 AIV infection markedly induces lncRNA-9101, which inhibits viral replication by positively regulating innate immunity. Mechanistically, lncRNA-9101 enhances the expression of MDA5 and TLR7, promotes IRF7 phosphorylation, elevates type I and III interferon production, strengthens STAT1 activation, ultimately leading to increased expression of Mx1 and OASL [28]. AIV, including H9N2, H5N1, and H7N9, upregulates a novel intronic circRNA-AIVR in host cells, which acts as a miRNA sponge to directly sequester miR-330-3p. miR-330-3p targets and degrades the mRNA of CREBBP, a key component of the IFN-β enhanceosome, and AIVR-mediated sequestration increases CREBBP expression, promoting IFN-β production and inhibiting multiple AIV subtypes [29]. For NDV, infection markedly upregulates miR-19b-3p in DF-1 cells. miR-19b-3p directly targets the 3′UTRs of RNF11 and ZMYND11, two negative regulators of NF-κB signalling, thereby enhancing NF-κB activation and promoting inflammatory cytokines and IFN-β production, ultimately inhibiting NDV replication [30]. Moreover, NDV infection also upregulates miR-375 in chicken tissues and DF-1 cells, which exerts dual effects. It directly targets the conserved M gene of NDV to degrade viral RNA and inhibit viral assembly/release. Meanwhile, it targets host ELAVL4 to accelerate cell cycle progression [31]. Furthermore, NDV infection upregulates host lncRNA-BST2-2 expression in an IFN/JAK/STAT-dependent manner, which directly interacts with the DNA-binding domain of IRF3, enhances the TBK1-IRF3 interaction, disrupts the IRF3-RACK1-PP2A inhibitory complex. These effects promote IRF3 phosphorylation and nuclear translocation, boosting type I interferon production and strengthening antiviral innate immunity [32]. NDV infection also reshapes circRNA expression profiles in DEFs, among which circRNA-FBXW7 (novel_circ_000094) is pronouncedly induced. Functional assays confirmed that overexpression of circRNA-FBXW7 significantly inhibits NDV replication, whereas its knockdown promotes viral proliferation. Bioinformatic analyses predict that circRNA-FBXW7 may function as a ceRNA by sponging miR-338-y and thereby modulating downstream target genes enriched in cell cycle and cellular senescence signalling pathways. This regulatory network may contribute to the restriction of NDV infection in ducks [33].

Beyond porcine and avian viruses, ncRNA-mediated antiviral defense is equally critical for ruminants, felines, and mink. BVDV strain NADL infection significantly upregulates miR-2411 expression in MDBK cells. It directly targets the 3′UTR of host Pelota mRNA to suppress its expression. Since Pelota acts as a proviral host factor essential for efficient viral protein synthesis, miR-2411-mediated Pelota inhibition markedly reduces BVDV replication [34]. BVDV NADL strain infection likewise markedly upregulates miR-29b expression in MDBK cells, which directly targets the 3′UTRs of autophagy-related genes ATG14 and ATG9A to suppress their expression. Given the dependence of BVDV replication on host autophagy, miR-29b-mediated autophagy inhibition pronouncedly limits viral proliferation [35]. For FHV-1, infection upregulates miR-101 expression in F81 cells in a cGAS-dependent manner. This miRNA directly targets the 3′UTR of SOCS5, a negative regulator of JAK-STAT signalling, to repress its expression. This results in enhanced type I interferon signalling, increased STAT1 phosphorylation, and elevated the expression of ISG15, Viperin, and IFITM1, potently restricting FHV-1 replication [8]. Similarly, FHV-1 infection upregulates miR-26a expression in F81 cells in a cGAS-dependent manner, which directly targets SOCS5 to reduce its expression, reinforcing type I interferon signalling and suppressing viral replication [36]. For MEV, infection significantly upregulates miR-320a and miR-140 in F81 cells. miR-320a directly targets the 3′UTR of feline TfR to suppress its expression and consequently impair viral entry, whereas miR-140 independently targets TfR to inhibit viral infection, and the two miRNAs act synergistically to restrict MEV infection [37]. These common induction mechanisms underline the conserved function of ncRNAs in interspecies antiviral immunity. These conserved antiviral ncRNA modules provide a foundational framework for understanding host defense programs against emerging viruses.

### Repression of host proviral ncRNAs

To mount an effective antiviral defense, animal hosts employ a universal and evolutionarily conserved strategy: transcriptionally or post-transcriptionally downregulating proviral ncRNAs that otherwise facilitate viral invasion, replication, or immune evasion. By reducing the abundance of these proviral ncRNAs, host cells relieve the suppression of innate immune signalling, restore interferon-dependent innate immune responses, and establish intracellular environment that limits viral propagation across diverse viral taxa.

Upon PRV-II infection, host cells implement a conserved antiviral strategy by downregulating proviral lncRNAs. Transcriptome sequencing reveals hundreds of differentially expressed lncRNAs in PK-15 cells infected with wild-type virulent PRV-II DX strain or attenuated gE/TK-deficient PRV-II strain, which are functionally enriched in interleukin secretion, metabolic processes, and axon extension. Among these, lncRNA-A02830 was identified as a key proviral factor. Silencing of lncRNA-A02830 significantly upregulates the transcription of IRF3, IFN-β, and Mx1, thereby activating type I interferon antiviral signalling and strongly inhibiting PRV-II replication. Conversely, overexpression of lncRNA-A02830 suppresses the IRF3/IFN-β/Mx1 axis and promotes viral proliferation. These findings confirm that host-mediated downregulation of proviral lncRNA-A02830 constitutes a critical innate immune mechanism against PRV-II infection by reinforcing interferon-dependent antiviral responses [9].

During infection with H5N1 highly pathogenic AIV and H1N1 avian-origin influenza viruses, host cells mount conserved antiviral responses via downregulation of the proviral miR-21-3p. miR-21-3p directly targets the 3′UTR of HDAC8 and suppresses its expression. In turn, HDAC8 acts as a critical antiviral regulator to restrict viral replication. Accordingly, ectopic expression of miR-21-3p promotes AIV replication by inhibiting HDAC8, whereas silencing HDAC8 mimics the proviral effect of miR-21-3p [38]. Consistent with these findings, infection with H5N1 highly pathogenic AIV significantly downregulates miR-21-3p expression in both patient sera and infected A549 cells. miR-21-3p acts as a proviral factor by directly targeting the 3′UTR of FGF2 and repressing its expression. Overexpression of miR-21-3p inhibits IFN-α/β signalling and the expression of downstream antiviral effectors (PKR, MxA, and OAS), thereby promoting the expression of viral proteins (M1, NP) and enhancing H5N1 replication. Conversely, restoring FGF2 expression reverses the proviral effect of miR-21-3p, reactivates the type I interferon response, and suppresses H5N1 proliferation [39]. In parallel, infection with H5N1 highly pathogenic AIV significantly downregulates miR-26a-5p expression in the lung tissues of resistant Ri chickens. As a proviral miRNA, miR-26a-5p directly targets the 3′UTR of MDA5 and acts as a translational repressor. Overexpression of miR-26a-5p suppresses the MDA5 signalling pathway, including downstream MAVS and IRF7, thereby reducing the expression of IFN-α, IFN-β, and the antiviral effector Mx1. The host’s downregulation of miR-26a-5p relieves MDA5 inhibition, restores innate antiviral signalling, and strengthens defense against avian influenza H5N1 in chickens [40]. Furthermore, infection with H5N1 and other RNA viruses significantly downregulates miR-340-5p expression in host cells. As a proviral miRNA, miR-340-5p directly targets the 3′UTRs of RIG-I and OAS2 and suppresses their expression. Overexpression of miR-340-5p attenuates RIG-I-mediated innate immune signalling, reduces the production of type I interferons and downstream antiviral genes, and markedly promotes H5N1 replication. In contrast, knockout or inhibition of endogenous miR-340-5p relieves the repression of RIG-I and OAS2, enhances antiviral immunity, and restricts the proliferation of H5N1 and other RNA viruses [41].

Beyond influenza viruses, NDV and other RNA virus infections trigger type I interferon signalling, which transcriptionally downregulates miR-1225-3p via the IFN/JAK/STAT pathway. miR-1225-3p acts as a proviral miRNA by directly targeting the 3′UTR of GAB3 to repress its expression. GAB3 is a positive antiviral adaptor by amplifying IFN-JAK-STAT signalling, promoting IRF-mediated interferon production, and enhancing the expression of IFIT1 and OAS1. Accordingly, host-mediated downregulation of miR-1225-3p relieves GAB3 inhibition, strengthens innate antiviral immunity, and restricts NDV replication [42]. For BVDV, infection significantly downregulates the host proviral lncRNA-IALNCR. LncRNA-IALNCR positively regulates MAPK8/JNK1 expression at both mRNA and protein levels. Its downregulation consequently reduces MAPK8/JNK1 expression, indirectly promoting the activation of caspase-3 and triggering cell-autonomous apoptosis, thereby antagonizing BVDV replication. Conversely, overexpression of lncRNA-IALNCR enhances MAPK8/JNK1 expression, inhibits apoptosis, and facilitates viral replication [10].

Collectively, these findings illustrate that targeted downregulation of proviral ncRNAs serves as a conserved and robust host defense strategy against multiple animal viruses. By relieving the inhibitory effects of proviral miRNAs and lncRNAs that dampen innate immunity, promote viral entry, or suppress apoptosis, hosts efficiently counteract viral immune subversion and establish a restrictive state against infection. Unifying principles derived from proviral ncRNA repression provide key insights for identifying antiviral targets against emerging viruses.

## Viral exploitation of host ncRNAs to facilitate viral replication

To establish productive infection, animal viruses actively hijack and exploit host ncRNA networks to suppress antiviral immunity, reshape cellular physiology, and create a replication-permissive environment. PEDV infection upregulates miR-218-5p expression, which directly targets the 3′UTR of HNRNPA3 and suppressing its expression. Reduced HNRNPA3 relieves its inhibitory effect on SREBF1 transcriptional activity via ZNF135, PI3K/AKT, and JNK-associated signalling pathways, thereby enhancing cellular lipid synthesis and promoting lipid droplet accumulation. This lipid metabolic remodelling generates a favourable microenvironment for the formation of viral replication complexes, ultimately promoting PEDV replication [43]. For PRRSV, infection significantly upregulates miR-142-5p, which directly targets the 3′UTR of FAM134B to reduce its expression. FAM134B functions as a key ER-phagy receptor, and its downregulation suppresses ER-phagy and causes ER expansion, thereby creating a favourable microenvironment for PRRSV replication. Furthermore, FAM134B-mediated ER-phagy promotes the recognition of viral PAMPs by TLR3/RIG-I, activates IRF3 phosphorylation and type I interferon signalling, and induces downstream antiviral ISG15, IFIT1, IFIT2, and IFIT3. Thus, the miR-142-5p/FAM134B axis enables PRRSV to simultaneously manipulate ER homeostasis and innate immunity to regulate PRRSV proliferation [44]. In addition, PRRSV infection also upregulates miR-216 in PAMs and CRL2843-CD163 cells via the p38 signalling pathway. miR-216 directly targets the 3′UTR of MAVS, a central adaptor in RIG-I/MDA5-mediated type I interferon signalling, to suppress its expression. Reduced MAVS abundance markedly inhibits IFN-β production and downstream antiviral ISGs (ISG15, IFIT1, and OAS1), thereby promoting PRRSV replication. Obviously, overexpression of MAVS abolishes the proviral effect of miR-216, supporting the miR-216/MAVS axis as a key immune evasion mechanism employed by PRRSV [45]. Similarly, PRRSV infection markedly upregulates miR-22 expression in MARC-145 cells. miR-22 directly targets the 3′UTR of HO-1, a key cytoprotective and antiviral host factor, and represses its expression at both mRNA and protein levels. Loss of HO-1 consequently diminished its antiviral effect, thereby markedly promoting PRRSV replication, whereas restoration of HO-1 expression counteracts the proviral activity of miR-22. These findings identify the miR-22/HO-1 axis as a novel mechanism through PRRSV hijacks host miRNA regulation to weaken cellular antiviral defenses [46]. PRRSV infection also significantly upregulates miR-24-3p in MARC-145 cells and primary PAMs. miR-24-3p also directly targets the 3′UTR of HO-1, a conserved cytoprotective and antiviral host factor, and suppresses its expression at both mRNA and protein levels. Downregulation of HO-1 relieves its antiviral effect, thereby markedly promoting PRRSV replication. In contrast, induction of HO-1 inhibits viral replication, and this effect can be fully reversed by overexpression of miR-24-3p. The miR-24-3p/HO-1 axis represents a key proviral mechanism by which PRRSV hijacks host miRNA to dampen host defense and facilitate viral proliferation [47]. Beyond targeting HO-1, PRRSV infection upregulates miR-30c expression while downregulating IFNAR2. Bioinformatics prediction and dual-luciferase reporter assays demonstrate that miR-30c directly targets the 3′UTR of IFNAR2, and mutations in either the target site or miR-30c seed region abolish this regulatory effect. miR-30c and IFNAR2 mRNA colocalize in the RISC. By targeting IFNAR2, miR-30c impairs type I interferon antiviral signalling and reduces the induction of antiviral ISGs, thereby promoting PRRSV replication [48]. In a parallel mechanism, PRRSV infection upregulates miR-30c expression in an NF-κB-dependent manner. miR-30c directly targets the 3′UTR of JAK1 to suppress its expression, thereby inhibiting type I interferon signalling, reducing STAT1 phosphorylation, and decreasing the induction of Mx1 and ISG15. This immune evasion strategy mediated by the miR-30c/JAK1 axis significantly promotes PRRSV replication both in vitro and in vivo, and miR-30c expression is positively correlated with viral load [49]. Moreover, PRRSV infection upregulates miR-373 expression by activating transcription factor Sp1, with the viral proteins Nsp9 and N implicated in this upregulation. miR-373 directly targets the 3′UTRs of NFIA, NFIB, IRAK1, IRAK4, IRF1, and IRF9, which act as key positive regulators of IFN-β production and signalling. By silencing these host factors, miR-373 suppresses IFN-β induction and downstream ISGs expression, thereby promoting PRRSV replication. Of note, NFIA and NFIB have been newly identified as antiviral proteins that directly activate IFN-β transcription and restrict PRRSV proliferation [50]. PRRSV infection also upregulates miR-376b-3p expression through the action of viral nonstructural proteins nsp4 and nsp11. miR-376b-3p directly targets the 3′UTR of TRIM22, a key antiviral restriction factor, and suppresses its expression. Under normal conditions, TRIM22 inhibits PRRSV replication by interacting with the viral N protein and promoting its lysosomal degradation through LC3-associated lysosomal trafficking. By silencing TRIM22, miR-376b-3p abolishes this antiviral effect and strongly promotes PRRSV replication [51]. Additionally, PRRSV infection upregulates miR-382-5p expression in MARC-145 cells. miR-382-5p directly targets the 3′UTR of HSP60 and inhibits its expression. HSP60 interacts with MAVS to promote IFN-β production and the expression of ISG15 and IFIT1 in a MAVS-dependent manner, thereby exerting potent anti-PRRSV activity. By silencing HSP60, miR-382-5p suppresses MAVS-mediated IFN-β induction and significantly promotes PRRSV replication [52]. PRRSV-2 infection significantly upregulates miR-541-3p in MARC-145 cells. miR-541-3p directly targets the 3′UTR of IRF7, a key transcription factor governing type I interferon production, and suppresses its expression. Reduced IRF7 abundance consequently attenuates IFN-β induction and downstream antiviral signalling, thereby promoting PRRSV-2 replication. On the contrary, overexpression of IRF7 restores IFN-β production and effectively inhibits viral replication [53]. In addition to miRNAs, PPRSV exploits lncRNAs to remodel host cellular barriers. PRRSV infection prominently induces the cytoplasmic lncRNA-103222771 in both Marc-145 cells and PAMs. Knockdown of lncRNA-103222771 markedly suppresses PRRSV replication. Mechanistically, lncRNA-103222771 negatively regulates the expression of CLDN4, a tight-junction protein that inhibits PRRSV entry by binding viral GP3. CLDN4 overexpression restricts PRRSV infection, while CLDN4 depletion promotes viral proliferation. Thus, the lncRNA-103222771/CLDN4 axis promotes PRRSV infection by dismantling the CLDN4-dependent cellular barrier, representing a novel pathogenic mechanism of PRRSV [54]. For PRV, infection significantly upregulates host lncRNA-000641 in 3D4/21, PK-15, and ST cells. LncRNA-000641 is predominantly distributed in the nucleus. Overexpression of lncRNA-000641 markedly promotes PRV replication, whereas its knockdown inhibits viral proliferation. Functionally, lncRNA-000641 suppresses IFN-α production and JAK-STAT1 phosphorylation, thereby dampening type I interferon-mediated antiviral innate immunity and creating a permissive environment for PRV replication [55]. For SVA, both infection and IFN-α stimulation significantly upregulate host nuclear lncRNA-ISL in PK-15 cells. LncRNA-ISL directly suppresses the initial transcription of antiviral G1P3 (IFI6) in a manner independent of JAK-STAT signalling and DNA methylation. G1P3 acts as a potent host restriction factor that inhibits SVA replication. Thus, the lncRNA-ISL/G1P3 axis represents a mechanism of viral immune evasion whereby SVA-induced lncRNA-ISL attenuates IFN-α-driven antiviral defense and facilitate viral infection [56]. Moreover, SVA infection significantly upregulates circRNA-8521 in PK-15 cells. CircRNA-8521 functions as a ceRNA that directly sponges miR-324, thereby relieving the inhibitory effect of miR-324 on its target gene LC3A. Upregulated LC3A promotes autophagy flux and facilitates SVA replication. The circRNA-8521/miR-324/LC3A axis represents another mechanism by which SVA hijacks host autophagy to promote infection [2]. These exploitative strategies enable efficient replication in porcine cells and tissues.

In avian species, AIV, ALV-J, and NDV also employ sophisticated ncRNA-centered strategies to subvert host defenses. H7N9 AIV infection significantly upregulates miR-664a-3p in A549 cells. miR-664a-3p directly targets and suppresses the host antiviral factor LIF, thereby promoting AIV replication. Conversely, inhibition of miR-664a-3p restores LIF expression, reduces viral titres by approximately 2 logs, and exerts broad-spectrum antiviral effects against multiple influenza A subtypes, including H1N1 and H3N2 [11]. H9N2 AIV infection also significantly upregulates lncRNA-8475 in DF-1 cells and chicken tissues. LncRNA-8475 promotes IAV replication by suppressing host innate antiviral immunity. Mechanistically, it downregulates TLR3/TLR7 expression, inhibits IRF7 phosphorylation and STAT1 activation, and consequently dampens downstream ISGs expression (e.g. Mx1, OASL, IFIT5). Knockdown of lncRNA-8475 reverses these effects and restricts viral proliferation [28]. In contrast, H9N2 AIV infection significantly upregulates nuclear-localized lncRNA-up4 in DF-1/HD11 cells as well as chicken tissues. Its expression is induced by viral RNA, poly(I:C), or LPS via the TLR3/MDA5-NF-κB pathway. Functionally, lncRNA-up4 restricts viral replication by suppressing type I interferon signalling, including IFN-β, Mx1, and OAS1, as well as ISRE activity [57]. For ALV-J, infection significantly upregulates miR-155 in CEF cells. Viral p27 protein binds and represses DDX3X transcription, promoting the processing of MIR155HG into mature miR-155. miR-155 directly targets the 3′UTRs of TRAF3 and STAT1, two core regulators of the type I interferon signalling, resulting in reduced IFN-β production, IRF7 activation, STAT1 phosphorylation, and downstream ISGs expression. This dual targeting enables ALV-J to effectively evade innate immunity and enhance viral replication. Thus, the p27-DDX3X-miR-155-TRAF3/STAT1 axis represents a distinctive viral immune evasion mechanism by ALV-J [58]. Similarly, ALV-J infection significantly upregulates miR-200b-3p in DF-1 cells. miR-200b-3p directly targets the 3′UTR of DUSP1 and inhibits its expression. DUSP1 acts as an antiviral host factor that restricts ALV-J replication, and knockdown of DUSP1 promotes viral proliferation, whereas overexpression suppresses it. The miR-200b-3p/DUSP1 axis promotes ALV-J replication by silencing the host antiviral factor DUSP1 [59]. ALV-J infection also significantly upregulates miR-23b in chicken spleen tissues and cells. miR-23b directly targets the 3′UTR of IRF1 and suppresses its expression. As a key antiviral factor, IRF1 promotes IFN-β production and inhibits ALV-J replication. Therefore, miR-23b-mediated repression of IRF1 reduces IFN-β production and substantially enhances viral replication. The miR-23b/IRF1 axis represents a key immune-evasion mechanism used by ALV-J [60]. Furthermore, ALV-J infection significantly upregulates miR-34b-5p in chicken immune tissues and DF-1 cells. miR-34b-5p directly targets the 3′UTR of MDA5, a key RLR antiviral receptor, and suppresses its expression. Deceased MDA5 inhibits the downstream IPS-1/IRF3/IFN-β signalling cascade and reduces the expression of antiviral effectors (PKR, OAS, Mx1, and MHC class I). Simultaneously, miR-34b-5p promotes cell proliferation, G1-S phase transition, cell migration, thereby creating a cellular state favourable for ALV-J replication and virion secretion. The miR-34b-5p/MDA5 axis represents a key immune-evasion mechanism for ALV-J [61]. For NDV, infection significantly upregulates miR-203a-3p in multiple human cell lines and PBMCs. miR-203a-3p directly targets the 3′UTRs of IFNAR1, JAK1, STAT1, and SOCS3, which are core components of the type I interferon signalling pathway. Simultaneous repression of these targets reduces ISRE activity and downstream ISGs expression, thereby substantially imparing host antiviral innate immunity. This immunosuppressive effect promotes NDV and influenza virus replication without affecting viral entry. The miR-203a-3p/IFN-I axis functions as a key immune homeostasis regulator and proviral factor for RNA viruses [62].

As a representative ruminant virus, BVDV exploits lncRNA networks to regulate immune signalling and endocytic trafficking for efficient replication. BVDV infection significantly upregulates lncRNA-CYLD in bovine cells. LncRNA-CYLD acts as a ceRNA that directly sponges miR-2383, thereby relieving the repression of CYLD by miR-2383. CYLD encodes a deubiquitinase that removes K63-linked polyubiquitin chains from RIG-I, consequently inhibiting RIG-I-mediated type I interferon signalling, IRF3 phosphorylation, and IRF3 nuclear translocation. This immune suppression strongly promotes BVDV replication. Conversely, lncRNA-CYLD knockdown or miR-2383 overexpression restores RIG-I signalling, enhances IFN-I production, and inhibits viral replication. These results suggest that the lncRNA-CYLD/miR-2383/CYLD regulatory axis is a key immune evasion mechanism of BVDV [63]. Additionally, BVDV infection significantly upregulates lncRNA-SMIM14 in MDBK cells. LncRNA-SMIM14 directly targets and positively regulates the endocytosis-related GTPase Rab3a, which colocalizes with endocytic regulators Rab5a and Rab7a to promote endocytic vesicle formation. Overexpression of lncRNA-SMIM14 or Rab3a markedly enhances BVDV replication, viral mRNA levels, progeny virus titres, and cytopathic effects; in contrast, their knockdown produces the opposite effects. These findings identify that the lncRNA-SMIM14/Rab3a axis represents a novel mechanism by which BVDV hijacks host endocytosis to facilitate its intracellular replication [64]. Collectively, these studies reveal that diverse animal viruses broadly exploit host ncRNAs to disable antiviral immunity, remodel cellular physiology, and construct a replication-permissive environment, highlighting ncRNAs as central hubs in viral pathogenesis and host-virus interactions. Conserved viral hijack strategies offer important implications for understanding pathogenic mechanisms of emerging viruses.

## Viral antagonism of ncRNA-mediated host antiviral responses

To overcome host restriction, animal viruses have evolved sophisticated strategies to specifically antagonize or downregulate antiviral ncRNAs that block viral entry, replication, and innate immune activation. For instance, PRRSV has developed multiple approaches to counteract miRNA- and lncRNA-mediated antiviral pathways in host cells. PRRSV infection markedly downregulates miR-122 in PAMs. miR-122 directly targets the 3′UTR of SOCS3 to repress its expression. As a key negative regulator of JAK-STAT signalling, SOCS3 constrains type I interferon production and thereby facilitate viral replication when depressed. Accordingly, PRRSV-mediated depletion of miR-122 increases SOCS3 expression, dampens the expression of ISG15 and IFIT2, and thereby promotes viral replication. Hence, the miR-122/SOCS3 axis constitutes a critical immune evasion strategy used by PRRSV to antagonize host antiviral defense [65]. PRRSV infection also significantly downregulates miR-181 in PAMs. miR-181 directly targets the 3′UTR of CD163, the essential cellular receptor required for PRRSV entry, and suppresses its expression, thereby blocking viral entry into host cells. By reducing miR-181 abundance, PRRSV relieves the repression of CD163, restores the availability of this entry receptor, and consequently facilitate viral entry and subsequent replication. The miR-181/CD163 axis therefore represents a host restriction mechanism antagonized by PRRSV to promote viral invasion [66]. Furthermore, PRRSV infection significantly downregulates miR-218 in PAMs. Similar to miR-122, miR-218 directly targets the 3′UTR of SOCS3 and represses its expression. Reduction of miR-218 expression consequently increases SOCS3 levels, resulting in attenuation of IFN-β and downstream ISGs expression, and thereby promoting viral replication. The miR-218/SOCS3 axis represents a key immune evasion mechanism by which PRRSV antagonizes host antiviral innate immunity [67]. Aside from miRNAs, PRRSV also counteracts antiviral lncRNAs. It significantly downregulates the evolutionarily conserved lncRNA-MAHAT in Marc-145 cells. MAHAT directly interacts with ZNF34 mRNA and recruits the RNA helicase DDX6 to form a complex that suppresses ZNF34 expression. Reduced ZNF34 relieves its inhibitory effect on type I interferon signalling, thereby enhancing IFN-α/β production and restricting PRRSV replication. By downregulating MAHAT, PRRSV elevates ZNF34 levels, dampens antiviral innate immunity, and facilitates viral proliferation. The MAHAT-DDX6-ZNF34 axis has been utilized by PRRSV [68].

In avian hosts, AIV, ALV-J, and NDV actively antagonize host antiviral ncRNAs to evade restriction. H5N1 significantly downregulates miR-1249 and miR-584-5p in A549 cells. Both miRNAs directly target the conserved coding region of the viral PB2 gene, suppress PB2 expression, and impair viral polymerase activity. This direct targeting mechanism restricts replication of multiple IAV subtypes. By reducing miR-1249 and miR-584-5p levels, H5N1 relieves PB2 inhibition, enhances viral replication, and consequently contributes to viral pathogenesis. Thus, the miR-1249/miR-584-5p-PB2 axis represents a critical host antiviral mechanism antagonized by H5N1 AIV [69]. H5N1 also significantly downregulates miR-18a-5p both in vitro and in vivo. miR-18a-5p directly targets the 3′UTR of NEDD9 and represses its expression. NEDD9 acts as a proviral factor that promotes H5N1 replication while suppressing IFN-β production. By reducing miR-18a-5p levels, AIV elevates NEDD9 expression, impairs antiviral innate immunity, and enhances viral replication and pathogenicity. The miR-18a-5p/NEDD9 axis represents a novel host antiviral pathway antagonized by H5N1 AIV [70]. Additionally, AIV, including H5N1, H1N1, and H9N2 subtypes, significantly downregulates the miR-30 family (miR-30a/b/c/d/e) in A549 cells. miR-30 directly targets the 3′UTRs of SOCS1, SOCS3, and NEDD4 to repress their expression. SOCS1/3 inhibit JAK-STAT signalling while NEDD4 degrades antiviral IFITM3. By reducing miR-30 levels, AIV elevates these negative regulators, impairs type I interferon responses, and promotes viral replication. The miR-30-SOCS1/3-NEDD4 axis represents a conserved host antiviral pathway that is antagonized by multiple AIV subtypes [71]. Furthermore, H5N1 significantly downregulates miR-324-5p both in vitro and in vivo. miR-324-5p exerts dual antiviral effects. It directly targets the conserved viral PB1 gene to degrade viral RNA and inhibit polymerase activity, and it represses host CUEDC2, a negative regulator of JAK1-STAT3 signalling, to enhance type I/III interferon and ISGs expression. By reducing miR-324-5p levels, H5N1 relieves suppression of both PB1 and CUEDC2, impairs antiviral immunity, and promotes efficient replication. Thus, the miR-324-5p-PB1-CUEDC2 axis represents a particular comprehensive host defense pathway antagonized by H5N1 AIV [72]. For ALV-J, its infection significantly downregulates the lncRNA-LTR5B in CEF and DF-1 cells. LncRNA-LTR5B is predominantly localized in the cytoplasm and directly binds to BiP (GRP78), sequestering Bip to prevent its translocation to the cell surface. BiP translocation is critical for ALV-J entry and pro-survival signalling in infected cells. By reducing lncRNA-LTR5B expression, ALV-J releases available BiP, promotes its cell-surface translocation, enhances viral entry, and suppresses apoptosis to facilitate viral replication. The lncRNA-LTR5B/BiP axis therefore represents an important host antiviral mechanism antagonized by ALV-J [73].

MEV similarly targets host antiviral miRNAs to evade restriction. MEV significantly downregulates miR-181b in F81 cells. miR-181b directly targets the coding region of the viral NS1 gene and represses NS1 protein translation, thereby inhibiting MEV replication. By reducing miR-181b levels, MEV relieves translational suppression of NS1, restores NS1-dependent viral replication, and facilitates its proliferation in host cells. Thus, the miR-181b/NS1 axis represents a key host antiviral mechanism antagonized by MEV [12]. In the infection model of canine-origin H3N2 CIV, miR-125b and miR-151, which are capable of suppressing viral replication, are markedly down-regulated upon infection, and the precise molecular mechanisms underlying this phenomenon remain to be further elucidated [74]. Collectively, these findings demonstrate that animal viruses employ diverse strategies to suppress, neutralize, or otherwise circumvent host antiviral ncRNA-mediated restriction. By disabling miRNA- and lncRNA-mediated restriction of viral entry, replication, and innate immune signalling, viruses efficiently overcome host defense and drive infection, pathogenesis, and persistence. Understanding viral antagonism of host antiviral ncRNAs facilitates the prediction of pathogenic mechanisms and intervention targets for future emerging viruses.

## ncRNA-mediated virus-host interplay: lessons from animal viruses for emerging viruses

The conserved regulatory paradigms of ncRNAs summarized in this review provide a unified conceptual framework for understanding and potentially predicting ncRNA-mediated mechanisms during infections with emerging animal viruses. To date, ncRNA-virus interactions have been well-characterized in a range of economically and epidemiologically important animal viruses, including SVA, H5/H7 subtype highly pathogenic AIV, PEDV, PRRSV, PRV, ALV-J, NDV, BVDV, FHV-1, CIV, and MEV. These models collectively establish fundamental principles of ncRNA-mediated host–virus interactions. Hosts establish multilayered antiviral defenses by inducing antiviral ncRNAs and repressing proviral ncRNAs whereas viruses counteract these defenses by hijacking host ncRNAs and antagonizing antiviral ncRNAs. Through these reciprocal regulatory processes, ncRNAs modulate antiviral innate immune signalling pathways, viral replication cycles, autophagy, apoptosis, and metabolic reprogramming for immune evasion and efficient proliferation. Nevertheless, the global regulatory landscape and underlying molecular mechanisms of ncRNAs remain poorly characterized in multiple epidemiologically critical emerging viruses.

Among established models, SVA and highly pathogenic H5/H7 AIV represent emerging RNA viruses with initially defined ncRNA-related mechanisms. Current evidence demonstrates that SVA exploits lncRNA-ISL to blunt interferon-dependent antiviral responses and regulates autophagy to promote replication via circRNA-8521 [2, 56]. In H5N1, H5N6, and H7N9 AIV, infection induces a diverse repertoire of antiviral ncRNAs, including lncRNA-45, lncRNA-61, and circRNA-AIVR, which are conservatively modulated in H5N1/H7N9, as well as miR-200c specifically upregulated in H5N6 and miR-203 specifically upregulated in H5N1, thereby initiating host antiviral defense [15, 25-27, 29]. Furthermore, H5N1 enhances host immune function by downregulating proviral ncRNAs such as miR-21-3p, miR-26a-5p, and miR-340-5p [38, 40, 41]. H7N9 suppresses antiviral immunity by hijacking miR-664a-3p, whereas H5N1 achieves immune evasion by antagonizing miR-1249, miR-584-5p, miR-18a-5p, the miR-30 family, and miR-324-5p [11, 69–72]. Nevertheless, compared with the relatively comprehensive regulatory network systematically summarized in this review, even in these emerging viruses with available preliminary data, several key regulatory mechanisms remain to be fully explored, including ncRNA-mediated regulation of apoptosis and lipid metabolic reprogramming, all of which warrant further investigation.

In contrast, our understanding of host ncRNA-mediated regulation of viral replication remains extremely limited for two important emerging pathogens, ASFV and SADS-CoV. To date, no studies have systematically characterized virus-induced differentially expressed ncRNAs, key regulatory targets, or molecular mechanisms by which these viruses exploit or antagonize ncRNA networks. Given these prominent knowledge gaps, the conserved regulatory principles established herein offer valuable insights for studies for investigating ncRNA-mediated host–virus interactions in ASFV and SADS-CoV. As diverse animal viruses converge on ncRNA-dependent modulation of interferon signalling, viral replication cycles, autophagy, apoptosis, and metabolic reprogramming, ASFV and SADS-CoV most likely employ evolutionarily conserved strategies to hijack proviral ncRNAs, antagonize antiviral ncRNAs, and remodel host cellular environments for replication and immune evasion. This predictive framework may greatly accelerate the identification of novel intervention targets.

Elucidating ncRNA-mediated crosstalk between emerging animal viruses and their hosts will be essential for comprehensively defining pathogenic mechanisms, developing broad-spectrum antiviral strategies, and strengthening preparedness for future outbreaks. For as-yet-unknown emerging viruses, ncRNA-centered regulation of innate immune signalling and viral replication cycles should represent important initial research priorities. Beyond these canonical frameworks, future studies should further investigate the roles of ncRNAs and expand our understandings of ncRNA-mediated regulation of autophagy, apoptosis, and metabolic reprogramming, thus deepening the understanding of pathogenic mechanisms of unknown emerging viruses and providing a theoretical basis for early warning and intervention. Another relatively underexplored area is the cGAS–STING pathway, a central host nucleic acid-sensing system that plays an important role in antiviral immunity [1]. Although numerous studies have established the importance of ncRNAs in regulating innate immune signalling, ncRNA-mediated regulation of the cGAS–STING pathway and its consequences for viral replication during animal virus infection remain comparatively poorly characterized. Systematic investigation of the interactions among miRNAs, lncRNAs, circRNAs, and the cGAS–STING axis may therefore represent an important direction for elucidating the pathogenic mechanisms of emerging viruses, particularly DNA viruses such as ASFV. Defining these regulatory relationships may also reveal previously unrecognized connections between ncRNA networks and cytosolic nucleic acid sensing.

Of note, systematic analysis in this review reveals that several ncRNAs (such as miR-155, miR-181, miR-203, miR-26a, and miR-30) are significantly differentially expressed and implicated in regulating viral replication across multiple animal viruses, suggesting that these molecules are not restricted to serving as regulators of a single virus, but rather represent core candidate targets with broad-spectrum regulatory potential across diverse viruses [7, 12, 23, 25, 36, 40, 48, 49, 58, 62, 66, 71]. Based on these conserved features, these aforementioned miRNAs could be prioritized as candidate targets for rapid screening and functional validation in SVA, H5/H7 subtype AIV, ASFV, SADS-CoV, and other future unknown emerging viruses. Such a strategy could accelerate the identification of conserved host dependency factors and antiviral restriction mechanisms and provide a rational basis for the development of broad-spectrum antiviral interventions. Importantly, the potential of these ncRNAs as broadly applicable therapeutic targets should be experimentally evaluated rather than inferred solely from their recurrence across different virus–host systems.

It should be noted that this review compiles experimental evidence across multiple host species, including swine, poultry, ruminants, felines, canines, and mink. There exists inherent inter-species divergence in baseline innate-immune landscapes (e.g. birds lack certain mammal-specific IFN subtypes, and cats exhibit distinct TLR repertoires relative to pigs), which can impose potential constraints on the evaluation of conservation within ncRNA-mediated regulatory networks [75, 76]. When performing cross-species comparisons or extrapolating the paradigms and candidate molecular targets summarized herein to emerging viruses, one must acknowledge the valid limits of cross-species comparison and refrain from over-interpreting the conservation of underlying regulatory mechanisms.

Collectively, this review systematically dissects the conserved regulatory paradigms of ncRNAs across a broad spectrum of animal viruses, providing critical insights for mechanistic investigations into emerging and uncharacterized viruses. This framework not only reveals the universal principles governing ncRNA-virus crosstalk, but also facilitates rational target identification, predictive modelling of pathogenesis, and the proactive design of countermeasures.

## Context-dependent duality of host ncRNAs: biological features and implications for antiviral target discovery

The preceding sections have systematically summarized three conserved regulatory paradigms: host antiviral defense, viral hijacking of host ncRNA networks, and viral antagonism of antiviral ncRNAs. Based on substantial experimental evidence, these paradigms provide a conceptual framework for understanding the general regulatory principles governing ncRNA-mediated host–virus interactions. Nevertheless, these paradigms represent simplified interpretations of complex experimental observations. Host ncRNAs do not possess invariable molecular properties. Compiled evidence from multiple animal-virus studies in this review indicates that several ncRNAs exhibit context-dependent duality: the same molecule can mediate either antiviral or proviral effects, and its ultimate functional output is jointly shaped by virus species, infectious dose, infection progression, and cell type. This functional duality manifests in two major forms: first, the same ncRNAs may exert opposing regulatory phenotypes under infection by distinct viruses; second, an ncRNA may undergo functional switching even during infection with the same virus when the surrounding cellular or infection context changes.

The divergent regulatory phenotypes of the same host ncRNA can be observed upon infection with different animal viruses, representing the most common manifestation of this duality. Members of homologous miRNA families can also exert drastically distinct biological effects across different species and virus infections. Take the miR-26 family as an example: miR-26a predominantly confers antiviral activity during PRRSV infection in pigs and FHV-1 infection in cats [7, 36]. By contrast, chicken-derived gga-miR-26a-5p displays proviral properties upon H5N1 AIV infection [40]. A similar functional divergence is observed for the miR-218 family. During PEDV infection, virus-upregulated miR-218-5p targets HNRNPA3 to remodel lipid metabolic pathways and acts as a proviral factor exploited by the virus [43]. During the PRRSV infection system, the homologous miR-218 targets SOCS3 to relieve repression of the type-I interferon pathway and thereby executes antiviral functions [67]. These observations demonstrate that sequence or family-level conservation of ncRNAs does not necessarily predict conservation of their functional consequences, which may instead depend on the specific virus–host regulatory network in which they operate. Beyond functional divergence across different viruses, duality of ncRNA effects can also be detected within infection by a single virus. miR-485 serves as a typical illustration of this phenomenon in the AIV infection model: at low viral doses, miR-485 mainly suppresses host antiviral immune responses and exhibits proviral properties; at high viral doses, however, miR-485 can directly target viral components and switch to exert prominent antiviral activity [77]. This suggests that conclusions drawn from in-vitro experiments using a single cell line under one defined infection condition cannot be directly extrapolated to authentic physiological states in-vivo tissues. When dissecting ncRNA regulatory networks of emerging viruses in future work, both infectious dose and temporal dynamics should be taken into account, and static interpretation of molecular functions ought to be avoided. Collectively, these examples warn against assigning rigid “antiviral” or “proviral” labels to any given ncRNA. When extrapolating the conserved paradigms summarized herein to decipher emerging animal viruses such as ASFV and SADS-CoV, one should anticipate potential functional inversion of homologous ncRNAs under novel viral backgrounds instead of mechanically adopting functional observations obtained from other experimental systems.

The context-dependent duality of host ncRNAs carries critical practical implications for screening broad-spectrum antiviral ncRNA targets based on the three conserved paradigms. Duality implies that robust antiviral activity observed for a molecule under certain experimental conditions does not guarantee stable protective effects across all biological contexts. Target evaluation should not rely solely on overexpression or knockdown assays performed in a single cell model; risks of functional switching originating from viral-strain variation, infectious-dose differences, and cell-type disparities must be adequately considered. When screening and evaluating candidate ncRNA-based interventions against emerging viruses, context-dependent duality should be treated as an important constraint, providing prerequisites for subsequent multi-dimensional functional validation and translational research.

## Concluding remarks and future perspectives

NcRNAs act as core regulatory molecules that shape the tug-of-war between animal viruses and their hosts. They participate in diverse key processes, including innate immune activation, viral replication cycle regulation, autophagy, apoptosis, and metabolic reprogramming through multilayered and multidimensional regulatory networks. This review systematically integrates experimental evidence from more than ten animal viruses across multiple species, including swine, poultry, ruminant, feline, canine, and mink hosts. On this basis, we identify three conserved regulatory modes: host antiviral defense, viral hijacking, and viral antagonism, clearly delineating the regulatory roles of host ncRNAs in virus-host interactions. Unlike studies that predominantly focus on single molecules or single viruses, this review integrates the differential expression (upregulation/downregulation) of ncRNAs upon infection with their actual functions (antiviral or proviral). We establish a cross-species and cross-virus conserved paradigm framework in animal virology, effectively addressing the prominent limitations of fragmentation and insufficient theoretical systems in current research. The regulatory mechanisms by which host ncRNAs modulate antiviral innate immune signalling pathways during animal virus infection are illustrated in Figures 2 and 3, whereas their modulations of host restriction factors, proviral host factors, viral replication cycles, autophagy, apoptosis and metabolic reprogramming are shown in Figures 4 and 5. The regulatory roles and molecular targets of individual host ncRNAs are systematically summarized in Tables 2 and 3.

**Figure 2.**
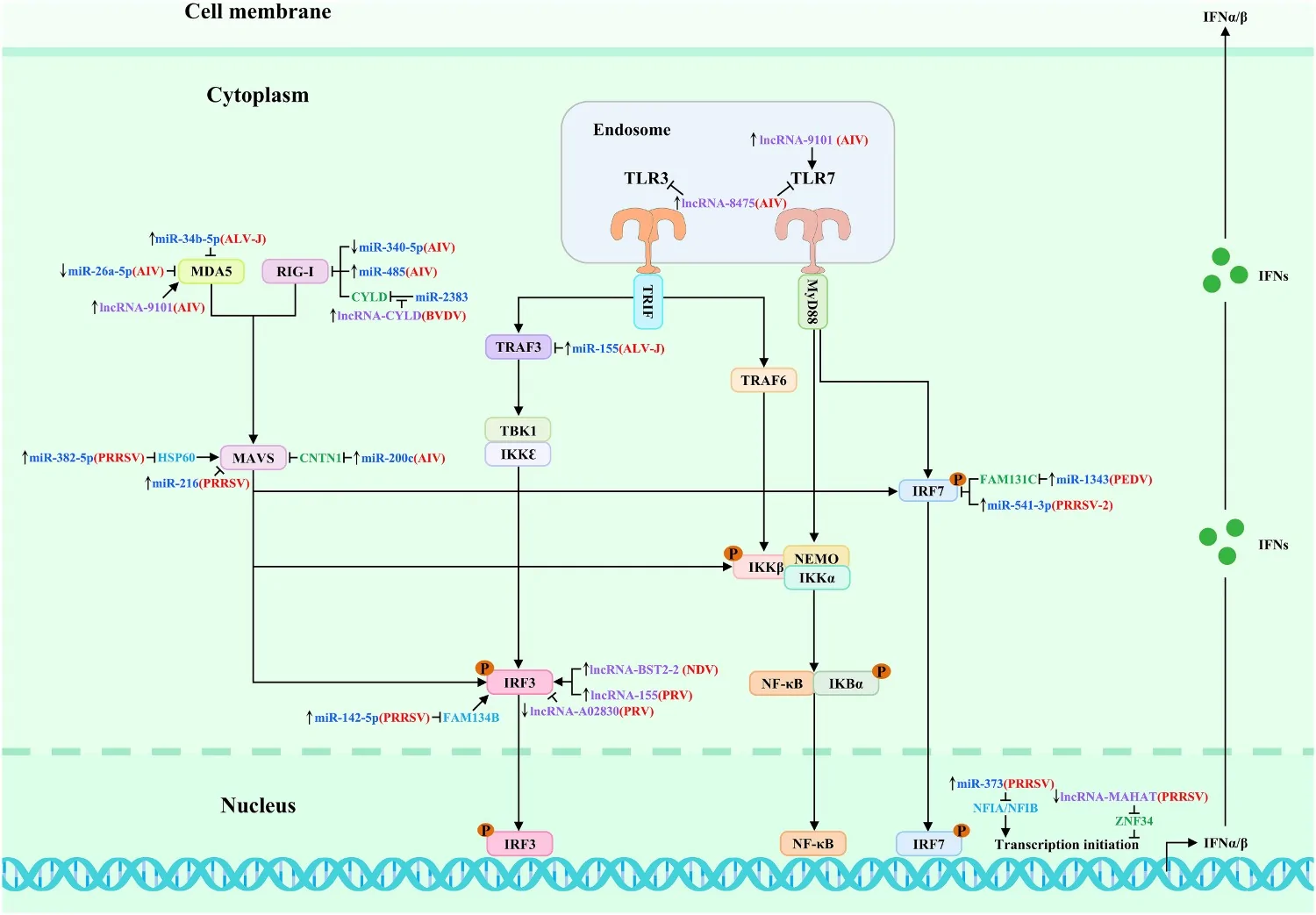
Regulatory roles of host ncRNAs in IFN-production-phase antiviral innate immune signalling during animal virus infection. Upon infection with animal viruses, host PRRs detect viral nucleic acids and trigger downstream innate immune signalling cascades, leading to the production of interferons and ISGs to establish an antiviral state. Host ncRNAs (including miRNAs and lncRNAs) are significantly upregulated or downregulated following viral infection, and they exert fine-tuned control over host antiviral responses and viral replication by targeting multiple key nodes of this signalling cascade, from PRR activation to interferon production. Representative ncRNAs labelled in this schematic, such as miR-26a-5p, miR-155, lncRNA-CYLD and lncRNA-BST2-2, exert either positive or negative regulation at multiple nodes of the signalling cascade, thereby tuning antiviral immune responses. Red indicates animal viruses, blue indicates miRNAs, purple indicates lncRNAs, green indicates proviral host factors, and light blue indicates host restriction factors.

**Figure 3.**
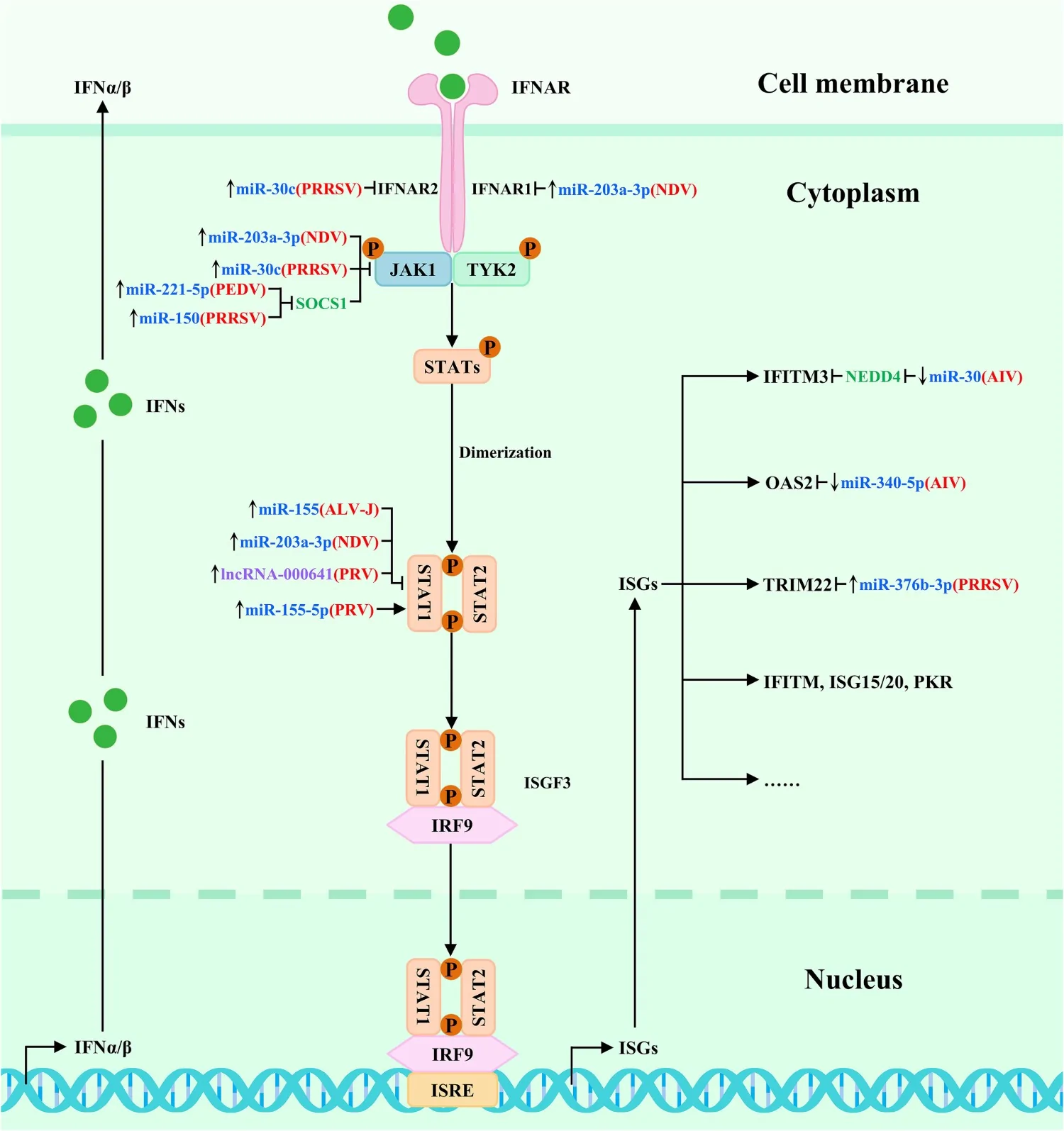
Regulatory roles of host ncRNAs in IFN-triggered JAK-STAT-ISG signalling cascades during animal virus infection. Following the secretion of interferons, IFN-α/β binds to membrane-localized IFNAR to initiate intracellular JAK-STAT signalling, which culminates in IRF9-ISRE-driven transcription of ISGs. Host-encoded miRNAs and lncRNAs modulate multiple steps within this cascade to shape downstream antiviral outputs. Representative ncRNAs labelled in this schematic, such as miR-30c, miR-203a-3p, miR-155 and lncRNA-000641, exert either positive or negative regulation at multiple nodes of the signalling cascade, thereby tuning IFN-dependent antiviral responses. Red indicates animal viruses, blue indicates miRNAs, purple indicates lncRNAs, and green indicates proviral host factors.

**Figure 4.**
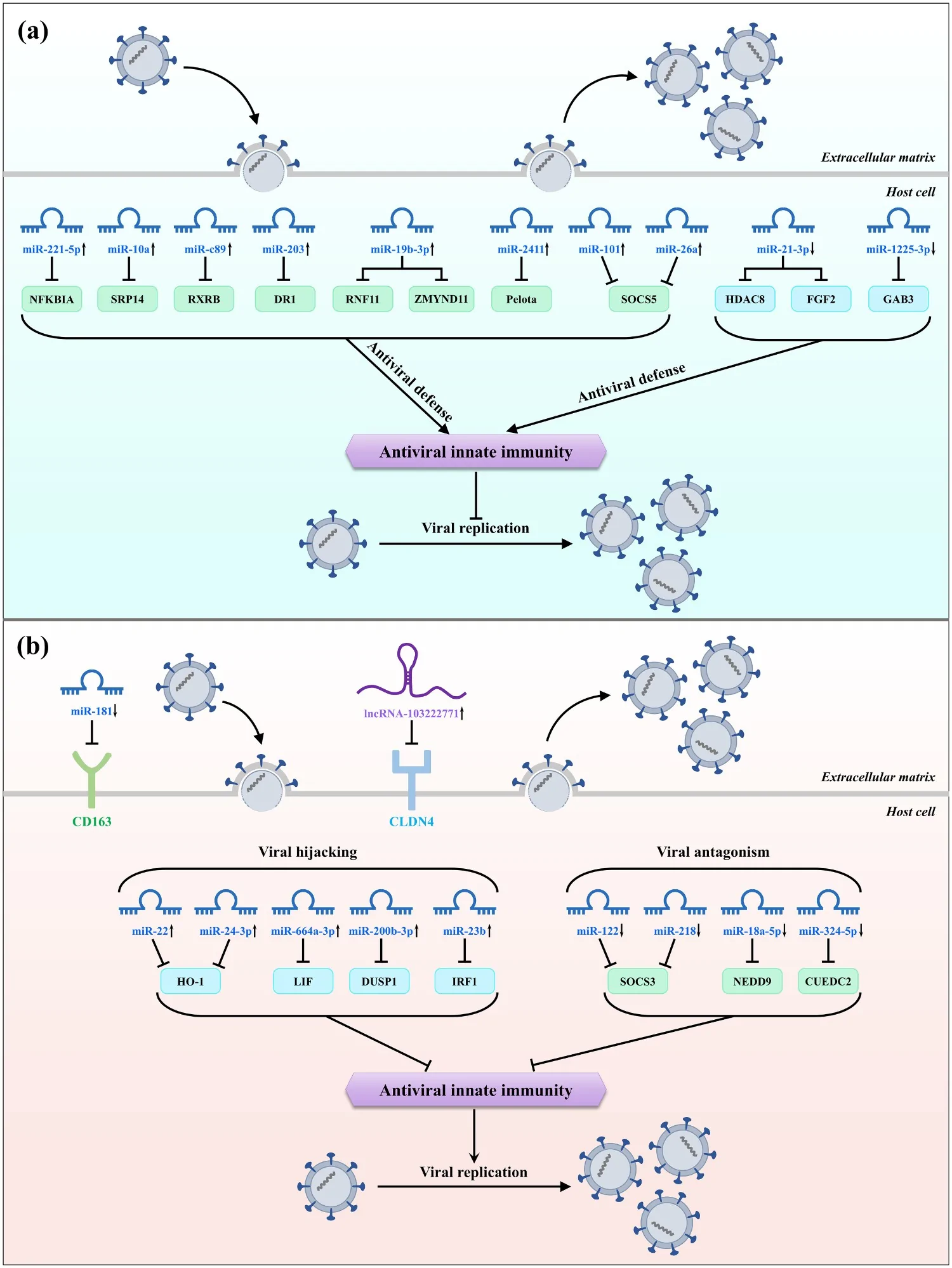
Schematic overview of core regulatory paradigms for host ncRNAs during animal virus infection. Panel (a) illustrates ncRNA-mediated antiviral defense: multiple host-derived ncRNAs target diverse host restriction factors and proviral host factors to potentiate antiviral innate immunity, which ultimately constrains viral replication. Panel (b) depicts two virus-favouring modes, namely viral hijacking and viral antagonism. Within these two paradigms, ncRNAs dampen antiviral innate immune responses and facilitate viral propagation. Representative ncRNAs labelled in this schematic, such as miR-221-5p, miR-181, miR-22 and lncRNA-103222771, exert either positive or negative regulation of antiviral immunity to shape viral infection outcomes. Blue indicates miRNAs, purple indicates lncRNAs, green indicates proviral host factors, and light blue indicates host restriction factors.

**Figure 5.**
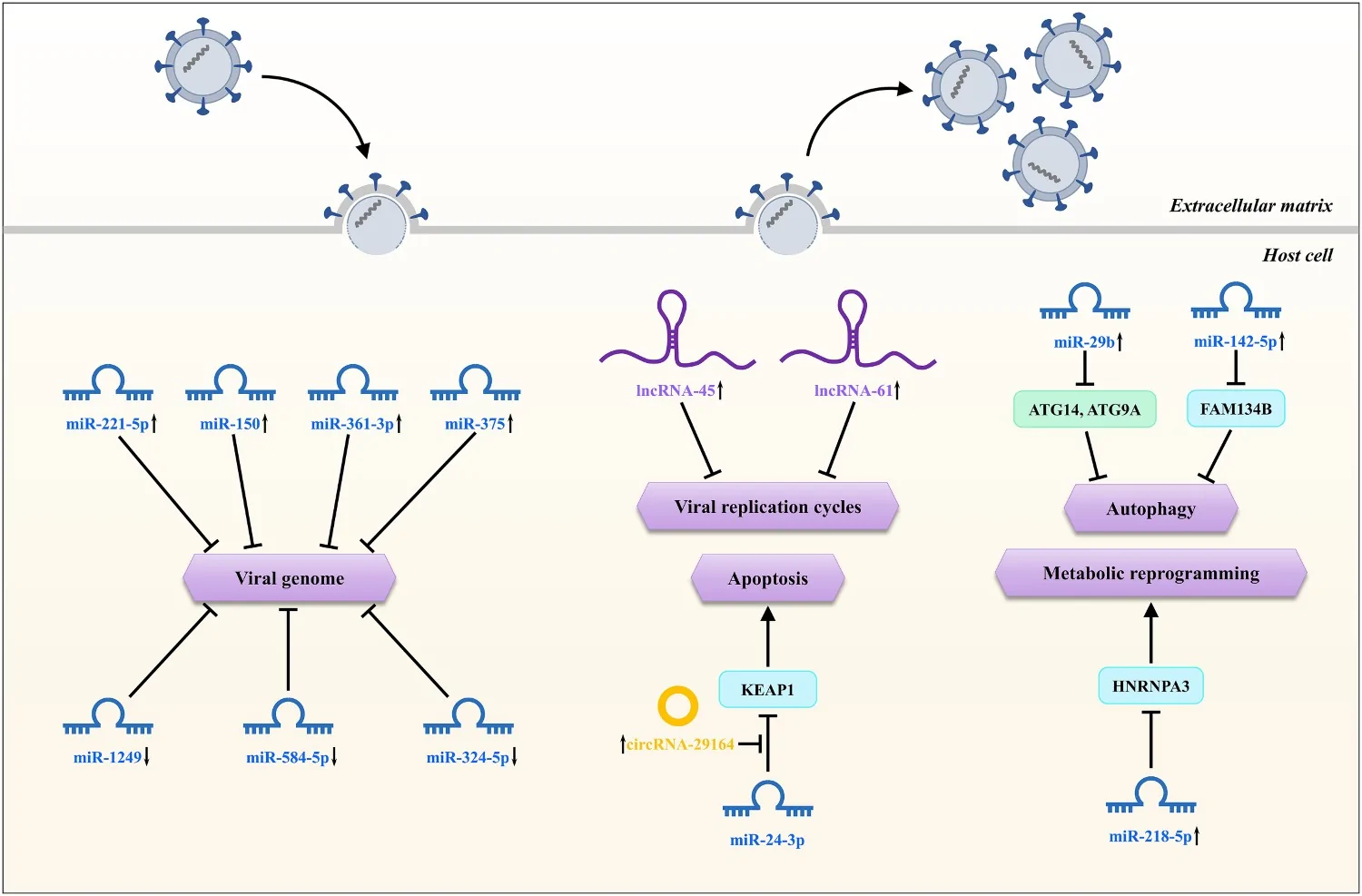
Diverse effector axes modulated by host ncRNAs during animal virus infection. Host-derived ncRNAs, including miRNAs, lncRNAs and circRNAs, govern multiple cellular and viral processes beyond core innate-immune signalling. These regulatory molecules directly target the viral genome, and modulate viral replication cycles, apoptosis, autophagy, and host metabolic reprogramming to shape the outcome of virus-host interactions. Representative ncRNAs labelled in this schematic, such as miR-221-5p, lncRNA-45, circRNA-29164, miR-29b, and miR-218-5p, exhibit regulatory activities across these distinct effector modules. Blue indicates miRNAs, purple indicates lncRNAs, orange indicates circRNAs, green indicates proviral host factors, and light blue indicates host restriction factors.

**Table 2.** Roles of host miRNAs implicated in modulating viral replication.

Viruses	MiRNAs	Targets	Effect on viral replication	Expression level after viral infection	Regulatory paradigms	Ref.
AIV	miR-1249	PB2, ↓	Inhibitive	↓	Viral antagonism	[69]
	miR-18a-5p	NEDD9, ↓	Inhibitive	↓	Viral antagonism	[70]
	miR-200c	CNTN1, ↓	Inhibitive	↑	Antiviral defense	[15]
	miR-203	DR1, ↓	Inhibitive	↑	Antiviral defense	[25]
	miR-21-3p	HDAC8, ↓	Promotive	↓	Antiviral defense	[38]
	miR-21-3p	FGF2, ↓	Promotive	↓	Antiviral defense	[39]
	miR-26a-5p	MDA5, ↓	Promotive	↓	Antiviral defense	[40]
	miR-30	SOCS1,SOCS3, NEDD4, ↓	Inhibitive	↓	Viral antagonism	[71]
	miR-324-5p	PB1, ↓ CUEDC2, ↓	Inhibitive	↓	Viral antagonism	[72]
	miR-340-5p	RIG-I, ↓ OAS2, ↓	Promotive	↓	Antiviral defense	[41]
	miR-485	RIG-I, ↓	Promotive	↑	Viral hijacking	[77]
		PB1, ↓	Inhibitive	↑	Antiviral defense	[77]
	miR-584-5p	PB2, ↓	Inhibitive	↓	Viral antagonism	[69]
	miR-664a-3p	LIF, ↓	Promotive	↑	Viral hijacking	[11]
ALV-J	miR-155	TRAF3, ↓ STAT1, ↓	Promotive	↑	Viral hijacking	[58]
	miR-200b-3p	DUSP1, ↓	Promotive	↑	Viral hijacking	[59]
	miR-23b	IRF1, ↓	Promotive	↑	Viral hijacking	[60]
	miR-34b-5p	MDA5, ↓	Promotive	↑	Viral hijacking	[61]
BVDV	miR-2411	Pelota, ↓	Inhibitive	↑	Antiviral defense	[34]
	miR-29b	ATG14, ↓ ATG9A, ↓	Inhibitive	↑	Antiviral defense	[35]
CIV	miR-125b	Not determined	Inhibitive	↓	Viral antagonism	[74]
	miR-151	Not determined	Inhibitive	↓	Viral antagonism	[74]
FHV-1	miR-101	SOCS5, ↓	Inhibitive	↑	Antiviral defense	[8]
	miR-26a	SOCS5, ↓	Inhibitive	↑	Antiviral defense	[36]
MEV	miR-140 miR-320a	TfR, ↓	Inhibitive	↑	Antiviral defense	[37]
	miR-181b	NS1, ↓	Inhibitive	↓	Viral antagonism	[12]
NDV	miR-1225-3p	GAB3, ↓	Promotive	↓	Antiviral defense	[42]
	miR-19b-3p	RNF11, ↓ ZMYND11, ↓	Inhibitive	↑	Antiviral defense	[30]
	miR-203a-3p	IFNAR1, ↓ JAK1, ↓ STAT1, ↓	Promotive	↑	Viral hijacking	[62]
	miR-375	M, ↓	Inhibitive	↑	Antiviral defense	[31]
PEDV	miR-1343	FAM131C, ↓	Inhibitive	↑	Antiviral defense	[6]
	miR-218-5p	HNRNPA3, ↓	Promotive	↑	Viral hijacking	[43]
	miR-221-5p	PEDV genome, ↓ NFKBIA, ↓ SOCS1, ↓	Inhibitive	↑	Antiviral defense	[13]
PRRSV	miR-10a	SRP14, ↓	Inhibitive	↑	Antiviral defense	[19]
	miR-122	SOCS3, ↓	Inhibitive	↓	Viral antagonism	[65]
	miR-142-5p	FAM134B, ↓	Promotive	↑	Viral hijacking	[44]
	miR-150	PRRSV genome, ↓ SOCS1, ↓	Inhibitive	↑	Antiviral defense	[20]
	miR-181	CD163, ↓	Inhibitive	↓	Viral antagonism	[66]
	miR-216	MAVS, ↓	Promotive	↑	Viral hijacking	[45]
	miR-218	SOCS3, ↓	Inhibitive	↓	Viral antagonism	[67]
	miR-22	HO-1, ↓	Promotive	↑	Viral hijacking	[46]
	miR-24-3p	HO-1, ↓	Promotive	↑	Viral hijacking	[47]
	miR-26a	Not determined	Inhibitive	↑	Antiviral defense	[7]
	miR-30c	IFNAR2, ↓	Promotive	↑	Viral hijacking	[48]
	miR-30c	JAK1, ↓	Promotive	↑	Viral hijacking	[49]
	miR-361-3p	PRRSV genome, ↓	Inhibitive	↑	Antiviral defense	[21]
	miR-373	NFIA/NFIB, ↓	Promotive	↑	Viral hijacking	[50]
	miR-376b-3p	TRIM22, ↓	Promotive	↑	Viral hijacking	[51]
	miR-382-5p	HSP60, ↓	Promotive	↑	Viral hijacking	[52]
	miR-541-3p	IRF7, ↓	Promotive	↑	Viral hijacking	[53]
	miR-c89	RXRB, ↓	Inhibitive	↑	Antiviral defense	[22]
PRV	miR-155-5p	Not determined	Inhibitive	↑	Antiviral defense	[23]

Note: miR-485 displays context-dependent duality with dose-dependent dual functions: proviral activity at low viral doses but antiviral activity at high viral doses; full mechanistic details are provided in Section 6.

**Table 3.** Roles of host lncRNAs and circRNAs implicated in modulating viral replication.

Viruses	LncRNAs/CircRNAs	Effect on viral replication	InfectionExpression	Regulatory paradigms	Mechanisms	Ref.
AIV	lncRNA-45	Inhibitive	↑	Antiviral defense	Inhibits viral polymerase and nuclear accumulation of NP/PA	[26]
	lncRNA-61	Inhibitive	↑	Antiviral defense	Interferes with viral entry, RNA synthesis and virion release	[27]
	lncRNA-8475	Promotive	↑	Viral hijacking	Downregulates TLR3/TLR7, inhibits IRF7/STAT1 activation	[28]
	lncRNA-9101	Inhibitive	↑	Antiviral defense	Enhances MDA5/TLR7 expression, promotes IRF7 phosphorylation	[28]
	lncRNA-up4	Promotive	↑	Viral hijacking	Suppresses type I IFN signalling and ISRE activity	[57]
	circRNA-AIVR	Inhibitive	↑	Antiviral defense	Sponges miR-330-3p, elevates CREBBP and IFN-β	[29]
ALV-J	lncRNA-LTR5B	Inhibitive	↓	Viral antagonism	Sequesters BiP to inhibit its nuclear translocation and viral entry	[73]
BVDV	lncRNA-CYLD	Promotive	↑	Viral hijacking	Sponges miR-2383 to upregulate CYLD, inhibits RIG-I signalling	[63]
	lncRNA-IALNCR	Promotive	↓	Antiviral defense	Upregulates MAPK8/JNK1 expression to inhibit apoptosis	[10]
	lncRNA-SMIM14	Promotive	↑	Viral hijacking	Positively regulates Rab3a to facilitate endocytic vesicle formation	[64]
NDV	lncRNA-BST2-2	Inhibitive	↑	Antiviral defense	Interacts with IRF3, boosts its phosphorylation and nuclear translocation	[32]
	circRNA-FBXW7	Inhibitive	↑	Antiviral defense	Putatively sponges miR-338-y to regulate relevant pathways	[33]
PEDV	lncRNA-446	Inhibitive	↑	Antiviral defense	Binds Alix to inhibit its ubiquitination and stabilize Alix protein	[18]
PRRSV	lncRNA-000397	Inhibitive	↑	Antiviral defense	Promotes antiviral ISGs expression to inhibit PRRSV replication	[14]
	lncRNA-103222771	Promotive	↑	Viral hijacking	Negatively regulates CLDN4 to disrupt cellular barrier	[54]
	lncRNA-MAHAT	Inhibitive	↓	Viral antagonism	Suppresses ZNF34 expression to boost type I interferon response	[68]
PRV	lncRNA-000641	Promotive	↑	Viral hijacking	Suppresses IFN-α production and JAK-STAT1 phosphorylation	[55]
	lncRNA-155	Inhibitive	↑	Antiviral defense	Enhances IRF3 activation to promote IFN-β production	[23]
	lncRNA-A02830	Promotive	↓	Antiviral defense	Suppresses IRF3/IFN-β/Mx1 signalling axis	[9]
	circRNA-29164	Inhibitive	↑	Antiviral defense	Sponges miR-24-3p to elevate KEAP1 and induce cell apoptosis	[24]
SVA	lncRNA-ISL	Promotive	↑	Viral hijacking	Suppresses G1P3 transcription to weaken antiviral defense	[56]
	circRNA-8521	Promotive	↑	Viral hijacking	Sponges miR-324 to upregulate LC3A and promote autophagy	[2]

Although a relatively systematic understanding has been achieved regarding the regulatory roles of host ncRNAs during animal virus infection, several fundamental questions remain unresolved. On the one hand, the regulatory mechanisms of host-encoded ncRNAs for major emerging animal viruses, such as ASFV and SADS-CoV, remain largely uncharacterized. The differential expression profiles, key regulatory targets, and core signalling axes of host ncRNAs have not been elucidated, which hinders rapid responses to newly emerging epidemics. On the other hand, most existing studies are limited to innate immune signalling and the viral replication cycle. The involvement of host ncRNAs in emerging regulatory layers, including peptide regulation, stress granule formation, epigenetic modification, and single-cell heterogeneity, remains insufficiently explored, and the completeness of the regulatory network needs to be improved [3, 78]. Furthermore, current studies are subject to multiple methodological constraints. Most evidence is derived from in-vitro cell systems, and conclusions may not translate to in-vivo conditions. When interpreting and extrapolating the conserved paradigms presented in Sections 2-4, the context-dependent duality of host ncRNAs described in Section 6 must be duly considered. The strength of evidence varies across different claims: some regulatory pathways are supported by multiple lines of experimental validation, whereas many candidate molecules lack rescue-assay confirmation. Discrepancies among published reports can occur, driven by experimental conditions. miRNA mimics, inhibitors and CRISPR-Cas13-based RNA-targeting tools carry off-target and specificity risks, which affect the interpretation of experimental data and translational research. Evaluation of ncRNA-virus interactions and candidate targets should therefore be prudent, and in-vitro observations should not be naïvely extrapolated to whole-animal settings [3]. Notably, the evolutionary origin underlying the context-dependent duality of host ncRNAs, as well as systematic comparisons between conserved and virus-specific regulatory mechanisms across animal virus families with distinct replication strategies, remain poorly understood and await further investigation with accumulating experimental evidence.

Although abundant functional data for host ncRNAs have been obtained from in-vitro cell-culture systems, only a small subset of molecules (miR-155-5p, lncRNA-155, miR-30c, miR-18a-5p, and miR-324-5p) have been functionally validated in animal infection models. Experimental evidence from large livestock and poultry animals remains scarce, and such in-vivo validation constitutes an essential prerequisite for the translational application of ncRNAs [23, 49, 70, 72]. Antiviral interventions based on ncRNAs for veterinary purposes are still confined to pre-clinical proof-of-concept stages, and no licensed ncRNA-derived veterinary antiviral therapeutics are currently available. Most intervention-oriented studies focus heavily on porcine virus models, where artificial microRNA (amiRNA) represents the predominant intervention modality; by contrast, therapeutic evaluations in avian and ruminant systems remain very limited. More broadly, general developmental frameworks for ncRNA-based antiviral therapeutics have been established, encompassing RNA engineering, LNP- or exosome-assisted delivery strategies. Nevertheless, multiple bottlenecks still need to be overcome, including the context-dependent duality of ncRNAs, delivery obstacles, scalable manufacturing costs, and safety assessment requirements for food-producing animals. Substantial additional pre-clinical investigations are therefore required before real-world deployment of ncRNA-based veterinary antiviral countermeasures [79, 80].

For future research, based on the conserved regulatory paradigm proposed herein, lessons from studies across different viruses can be adopted to extrapolate validated core axes and potential broad-spectrum antiviral targets to newly emerging viruses, enabling efficient screening of critical targets and in-depth dissection of pathogenic mechanisms [1, 3, 4]. Furthermore, the regulatory scope of host ncRNAs should be expanded to focus on underexplored frontiers, including peptide regulation, stress granule formation, epigenetic modification, and single-cell heterogeneity, toward constructing a more comprehensive regulatory landscape of virus-host interactions. Meanwhile, broadly conserved molecules such as miR-155, miR-181, miR-203, miR-26a, and miR-30 should be prioritized for rapid validation of their regulatory roles in emerging viruses, to define their potential as candidate targets with broad-spectrum regulatory potential. Furthermore, emerging technical approaches such as multi-dimensional transcriptomics (including single-cell and spatial transcriptomics) and RNA-targeted editing tools exemplified by CRISPR-Cas13 offer substantial opportunities for research in this field. These powerful techniques remain underutilized in studies of ncRNA-mediated interplay between animal viruses and hosts. Deployment of these methodologies enables the dissection of cell-type-specific ncRNA responses, reconstruction of high-resolution ncRNA regulatory networks, and high-throughput screening and validation of functional ncRNA candidates, opening productive avenues for future investigations [80].

In the long term, beyond host ncRNAs, it is critical to continuously and deeply dissect the virus-host interplay mediated by various host biomolecules, including proteins, carbohydrates, lipids, hormones, and inorganic salts [3]. Such efforts will not only refine the fundamental theoretical framework of animal virus pathogenesis but also provide a holistic research pipeline for emerging animal viruses, covering mechanistic dissection, target mining, and intervention strategies. Grounded in the three core regulatory modes (host antiviral defense, viral hijacking, and viral antagonism), the theoretical framework established in this review can offer theoretical guidance for addressing unknown livestock and poultry viral epidemics, and provide a scientific basis for developing novel antiviral interventions and enhancing disease prevention and control in the livestock industry.
